# Expanded algal cultivation can reverse key planetary boundary transgressions

**DOI:** 10.1016/j.heliyon.2018.e00538

**Published:** 2018-03-01

**Authors:** Dean Calahan, Edward Osenbaugh, Walter Adey

**Affiliations:** a Department of Botany, National Museum of Natural History, Smithsonian Institution, Washington, DC 20013, USA; b Softforce, Inc., Gilbert, IA 50105, USA

**Keywords:** Environmental Science

## Abstract

Humanity is degrading multiple ecosystem services, potentially irreversibly. Two of the most important human impacts are excess agricultural nutrient loading in our fresh and estuarine waters and excess carbon dioxide in our oceans and atmosphere. Large-scale global intervention is required to slow, halt, and eventually reverse these stresses. Cultivating attached polyculture algae within controlled open-field photobioreactors is a practical technique for exploiting the ubiquity and high primary productivity of algae to capture and recycle the pollutants driving humanity into unsafe regimes of biogeochemical cycling, ocean acidification, and global warming. Expanded globally and appropriately distributed, algal cultivation is capable of removing excess nutrients from global environments, while additionally sequestering appreciable excess carbon. While obviously a major capital and operational investment, such a project is comparable in magnitude to the construction and maintenance of the global road transportation network. Beyond direct amelioration of critical threats, expanded algal cultivation would produce a major new commodity flow of biomass, potentially useful either as a valuable organic commodity itself, or used to reduce the scale of the problem by improving soils, slowing or reversing the loss of arable land. A 100 year project to expand algal cultivation to completely recycle excess global agricultural N and P would, when fully operational, require gross global expenses no greater than $2.3 × 10^12^ yr^−1^, (3.0% of the 2016 global domestic product) and less than 1.9 × 10^7^ ha (4.7 × 10^7^ ac), 0.38% of the land area used globally to grow food. The biomass generated embodies renewable energy equivalent to 2.8% of global primary energy production.

## 1 Introduction

The resource demands of Earth's population, which may exceed eleven billion people by 2100 (United Nations, 2017), will likely continue to intensify stresses on ecosystem services (Boyd and Banzhaf, 2007; World Resources Institute & UNICEF, 2005). Among the most pressing threats is transgression of key planetary boundaries by overloading of the global N and P biogeochemical cycles via excess agricultural fertilizer/manure application and incompletely treated sewage (Bouwman et al., 2005, 2013; Boyer et al., 2006; Carpenter et al., 1998; MacDonald et al., 2011; Moe and Rheingans, 2006; Potter et al., 2010; Sims et al., 1998; Steffen et al., 2015). Fresh and marine waters receiving these excess nutrients are experiencing harmful algal blooms (HABs) and their concomitant negative effects on ecosystems and human health with increasing frequency and extent (Fu et al., 2012; Glibert et al., 2005; Hallegraeff, 1993; Heisler et al., 2008). Additionally, unsustainable fossil fuel extraction and combustion are overloading the global C biogeochemical cycle, warming our climate and acidifying our oceans (Ciais et al., 2014; Mostofa et al., 2015). Human activity and these biogeochemical cycles are tightly coupled with photosynthesis, as primary production fixes environmental N, P, and C into biomass (Field et al., 1998). Thus if photosynthesis could be greatly expanded globally, and the resulting biomass effectively managed, stresses on multiple Earth systems could be relieved. With a sufficiently large increase in primary production, negative impacts on N and P cycling could be stabilized and eventually reversed, with concomitant major relief of stress on C cycling.

As N, P, and C cycle globally, and their chemical compounds become dilute and pervasive once released into the environment, projects to appreciably sequester or recycle them need to be globally distributed at large scale. One practice intended to mitigate dilute N and P pollution is the constructed treatment wetland (CTW) (Hoffmann et al., 2011; Kivaisi, 2001; Vymazal, 2010). CTWs embed some excess P in the soil while volatilizing much of the excess N, and to a lesser extent sequester these nutrients as biomass, with effective P removal requiring increased complexity and expense (Keller and Knight, 2004; Fasching et al., 2015). Due to linkage with photosynthesis, CTWs also sequester some C in biomass. While low operating costs are claimed for CTWs, these estimates have not generally included long term harvesting to counter maturation and degradation (Keller and Knight, 2004); a multi-decadal harvest cycle requires major expense for dredging, transportation and sequestering or processing the spoil. Further advantages ascribed to CTWs include increased biodiversity, mitigation of pathogens and additional pollutants, and applicability over a range of geographical, climatic, and socio-economic environments (Semeraro et al., 2015; Kivaisi, 2001; Zhang et al., 2015). However, the areal effectiveness of CTW nutrient removal tends to be so low that treating an agricultural basin to pre-industrial levels of N and P with would require converting a substantial proportion of a basin, possibly including crop growth areas, to wetlands. The floating treatment wetland (FTW), an aquatic analog of the CTW, imposes additional operational burdens, is potentially viable only in relatively calm waters - greatly limiting its applicability - and can increase, rather than decrease, nutrient loading if not managed properly (Stewart et al., 2008; Wang and Sample, 2014; Winston et al., 2013). If expanded to a size functional for treating a major lake or estuary, a FTW would likely interfere with other commercial and recreational uses.

Where concentrated pollutants are released, as from sewage treatment plants or factories, effective industrial treatment is feasible but expensive, and is actively, if sporadically, performed mostly in wealthy countries (Elimelech, 2006; Environmental Protection Agency, 2007, 2015; Høibye et al., 2008). However, even with cleanup of concentrated N and P via expanded industrial treatment, agriculture unavoidably produces global nonpoint N and P pollution from application of fertilizer and manure that infiltrates nearby surface and subsurface waters, with septic systems and cesspools contributing additional N and P in much of the world (Cairncross, 2003; Ribaudo et al., 2001). Beyond cleanup of excess N and P, CO_2_-specific solutions that compress or chemically fix this pollutant from industrial facilities have poor economics and depend on storage in sites of uncertain stability (Cuéllar-Franca and Azapagic, 2015; Esteves and Morgado, 2012). Fortifying the oceans with limiting micronutrients such as iron could remove large quantities of macronutrients (Boyd et al., 2000; Buesseler et al., 2008; Naqvi and Smetacek, 2011) by stimulating large plankton blooms that then sink, but the long-term impacts of this approach are poorly understood, and the loss of potentially valuable biomass weakens the economics. It is essential to seek more affordable and effective solutions.

**Fig. 1:**
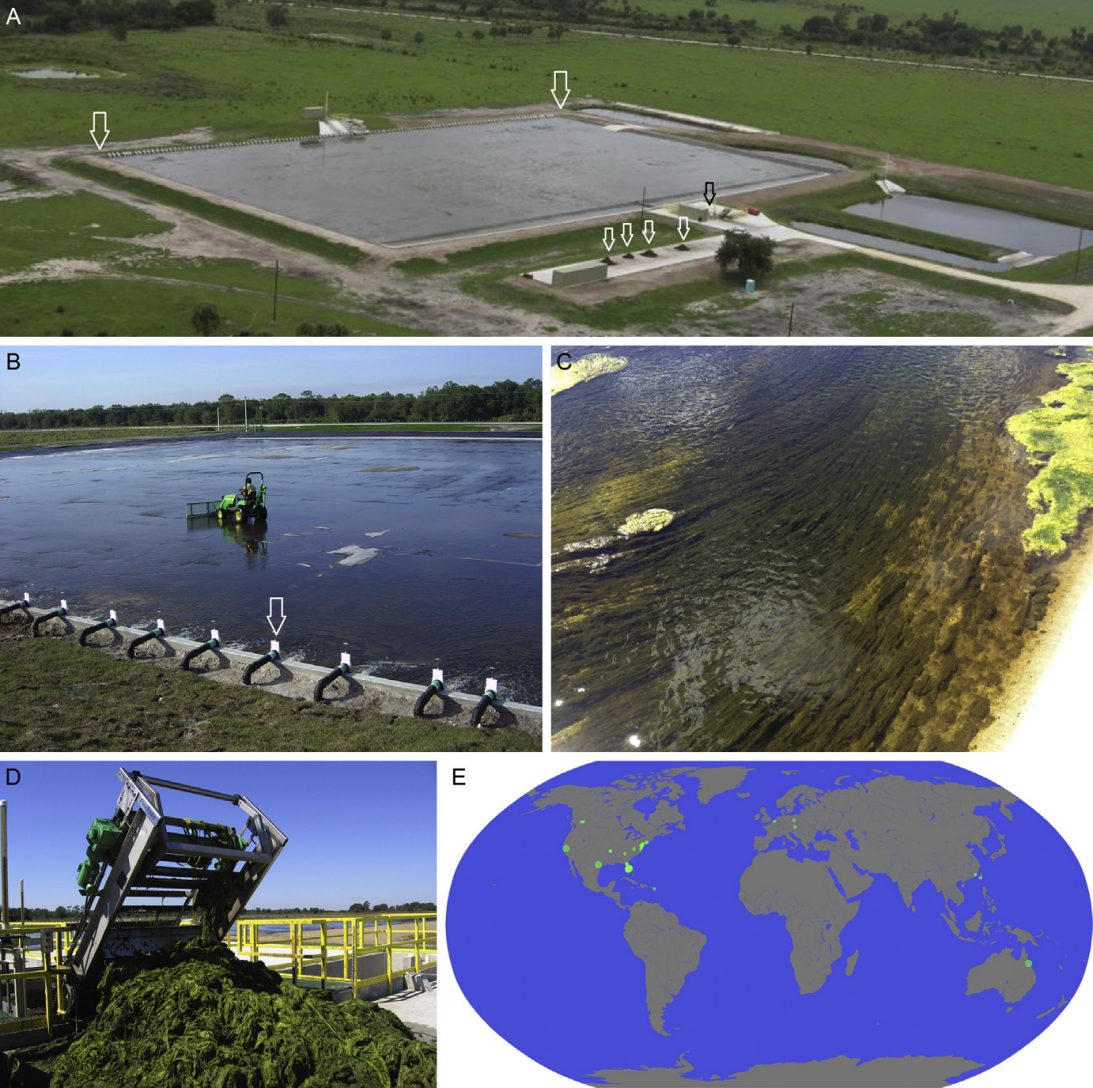
Algal Turf Scrubbing (ATS). (A) Aerial view of a ∼1 ha (∼2.5 ac) system in Florida. Water surges onto the floway along the upper contour, whose extrema are indicated by large white arrows. Piles of dewatered (∼8% solids) harvested biomass are indicated by small white arrows. Small black arrow indicates a sump into which biomass is scraped for harvest. (B) Harvesting using a small agriculture vehicle equipped with a blade for scraping. Scraped material is pushed into the sump indicated by the solid arrow in A. Note typical input surger (white arrow). (C) Person's-eye view of algal biomass ready for harvest. (D) Collecting gravity-dewatered harvest from sump using a motorized rake. (E) Locations of past and present outdoor ATS pilot projects (small circles) and production systems (large circles).

Here we evaluate the capability of the algal cultivation practice known as algal turf scrubbing (ATS) (Stewart, 2004; Adey et al., 2011) to intervene in humanity's overloading of the N, P, and C biogeochemical cycles. One of us (Adey) is the inventor of this process, a reliable technology that harnesses rapidly growing attached algal polycultures, also known as periphyton, confined within an open-field photobioreactor, to photosynthetically remove N, P, C, and other aquatic pollutants from point and nonpoint sources in both fresh and marine waters (Adey et al., 1993, 1996; Mulbry et al., 2010; Rothman et al., 2013). This practice also oxygenates the cleaned effluent, increases its pH, and produces copious biomass. ATS is a trademark, and Algal Turf Scrubber a registered trademark, of Ecological Systems Inc., the primary R&D entity for the technology; these are licensed to HydroMentia Technologies LLC. ATS has been deployed at hectare scale for decades, with low capital and operational expenses (CapEx and OpEx) compared to other nonpoint treatment solutions, demonstrating reliable operation and productivity equal to or better than local high-performance agriculture. ATS introduces what is essentially a new agricultural crop: nonspecific algal biomass, low in water consumption and independent of the extractive industries producing fertilizer and other commodities essential for the highest agricultural productivities. ATS has been shown at more than forty locations to polish water with already low nutrient concentrations, treat secondary sewage as well as raw and digested animal manure, recycle aquaculture/mariculture wastewater, remove nutrients from dredge spoil, and clean agricultural runoff (Fig. 1E, Table 1). Mean annual ash-free dry weight (AFDW) productivity in temperate and sub-tropical zones routinely exceeds 55 t ha^−1^ yr^−1^ (20 T ac^−1^ yr^−1^); recent work supports the likelihood of increasing productivity, with annual AFDW means of 150 t ha^−1^ yr^−1^ (54 ton ac^−1^ yr^−1^) published (Adey et al., 2013). A typical production-scale facility occupies ∼1 ha, treating ∼4.0 × 10^7^ L d^−1^ (∼1.1 × 10^7^ gal d^−1^), with larger volumes handled using multiple instances of a modular design. Expenses can vary; for CapEx a range of $2.5 × 10^5^ ha^−1^ ($1.0 × 10^5^ ac^−1^) is reasonable where minimal site preparation is required, increasing to $1.0 × 10^6^ ha^−1^ ($4.0 × 10^5^ ac^−1^) with complex site preparation. OpEx can range from $4.0 × 10^4^ ha^−1^ yr^−1^ ($1.6 × 10^4^ ac^−1^ yr^−1^) for single-pass operation to $7.0 × 10^4^ ha^−1^ yr^−1^ ($2.8 × 10^4^ ac^−1^ yr^−1^), for more sophisticated operations (Hoffman et al., 2017). All monetary values represent approximate 2018 US dollars.

**Table 1:** Locations of outdoor ATS and similar studies and production-scale facilities (see also Fig. 1E).

long	lat	citation
146.8	−19.3	Adey and Loveland (2007)
−80.5	26.7	Adey et al. (1993)
−74.5	40.4	Adey et al. (1996)
−76.3	37.9	Adey et al. (2013)
−120.9	50.8	Bothwell (1983)
−119.7	50.8	Bothwell (1989)
−64.7	17.4	Carpenter et al. (1991)
120.3	23.0	Chang et al. (2011)
117.8	24.5	Chen et al. (2015)
−121.1	37.5	Craggs et al. (1996)
−80.4	27.4	D'Aiuto et al. (2015)
−80.4	37.2	Huang et al. (2013)
−79.0	36.0	HydroMentia (2017)
−80.9	27.2	HydroMentia (2013)
−82.0	27.9	HydroMentia (2013)
−81.9	26.7	HydroMentia (2017)
−80.9	27.3	HydroMentia (2017)
−80.9	27.5	HydroMentia (2017)
−80.4	27.6	HydroMentia (2017)
−80.4	27.6	HydroMentia (2017)
−80.5	27.6	HydroMentia (2017)
−81.4	28.6	HydroMentia (2017)
−82.5	29.9	HydroMentia (2017)
−85.0	34.8	HydroMentia (2017)
−76.5	39.2	HydroMentia (2017)
−76.5	39.2	HydroMentia (2017)
−76.3	39.8	HydroMentia (2017)
−73.8	40.6	HydroMentia (2017)
3.6	51.1	Liu et al. (2016)
16.4	48.2	Mayr et al. (2015)
146.8	−19.3	Morrissey et al. (1988)
−76.9	39.0	Mulbry et al. (2008)
−76.7	38.8	Mulbry et al. (2010)
−76.5	39.1	Mulbry et al. (2010)
−76.3	39.4	Mulbry et al. (2010)
−76.2	38.6	Ray et al. (2015)
−76.4	37.2	Rothman et al. (2013)
−94.2	36.2	Sandefur et al. (2011)
−94.1	36.1	Sandefur et al. (2014)
−82.3	29.6	Sindelar et al. (2015)
−98.8	29.5	Stewart (2004)
16.2	51.6	Sukačová et al. (2015)
−103.3	34.2	Yan and Arellano (2015)
−75.9	39.0	Kangas and Mulbry (2014)

An ATS facility is characterized by a shallow, flat, inclined (0.5–2% slope), exposed surface (the floway) (Fig. 1A), either roughened or lined with a high surface-area fabric to provide a growth substratum. A wave- or pulse-generator directs influent into the floway along its upper contour (Fig. 1A, B), producing a shallow (2–5 cm (0.7–2 in) deep) flow coursing downslope. Cells of diverse algal taxa, ubiquitous in surface waters, adhere to the substratum and rapidly propagate, maturing into a community of attached filaments and trapped cells (Fig. 1B, C). Cell growth fixes dissolved N, P, C, and other solutes and particles into the developing biomass, removing them from the influent. Weekly to biweekly harvest removes the majority of the biomass, staging the remnant algal cells for another production cycle. Continuous operation ensures perpetual sampling of the influent for new algal species and strains, increasing the facility's biodiversity over time. Typical mature ATS communities have diversities exceeding 100 species (Kangas et al., 2017). Pulsing imposes turbulent flow, increasing light penetration and mixing, allowing even dilute nutrient sources to support high productivity (Adey and Steneck, 1985). Photosynthesis drives the buffer system to high pH via CO_2_ and HCO_3_^−^ uptake, and supersaturates the effluent with O_2_. If present, heavy metal ions are incorporated into the biomass, and many toxic organic molecules are removed or detoxified (Adey et al., 1996).

Typically, ATS operates in a single-pass mode, with influent traversing the floway once before returning to its source. Multiple passes may instead be performed, either with the effluent from one floway used as the influent for another, emulating a single longer floway, or by recirculating water through the same floway multiple times before discharge. Single-pass mode requires less CapEx and OpEx than more complex operational modes, but can accomplish less total nutrient reduction, as carbon limitation, arising along the floway due to removal of this nutrient as it is incorporated into biomass during photosynthesis, reduces areal productivity and thus nutrient uptake. Introducing CO_2_ at intervals along the floway can reverse carbon limitation, increase areal productivity, and provide the capability to reduce nutrient loading to oligotrophic levels. Photosynthesis adds O_2_ and drives the buffer system towards its higher-pH, CO_3_^2−^ dominated profile, upgrading the water (where hypoxia and/or acidification is an issue) beyond simply removing pollutants. Discharge of higher pH water can potentially be an issue. However, the HCO_3_^−^/CO_3_^2−^ buffer strength is inherently low, and in this case dilute with most of the HCO_3_^−^ removed, so a final treatment step of mixing it with untreated input water or introducing CO_2_ may be desirable in some circumstances. These considerations suggest design and operation optimizations for specific situations. For N-limited basins, a subset of the floway area can be operated for P recovery by using floways sufficiently long to drive pH above 8.5 to chemically precipitate phosphate salts (Adey et al., 1993; Craggs et al., 1996). For P limited basins, there is no correspondingly simple operation to precipitate surplus N-containing salts. However, some of the biomass from these basins could be processed to extract P that could be used to supplement the N-rich influent.

Because the effectiveness of an ATS facility at removing N and P pollution correlates with its biomass productivity, the fate of the biomass is an essential consideration for project planning, ideally producing revenue to help offset treatment costs. Biomass produced from waters with low toxin concentrations could be used in agriculture as a fertilizer/soil amendment (Mulbry et al., 2005, 2007), as a source of compost (Shiralipour and Epstein, 2005), or as an animal or fish feed (Adey and Purgason, 1998a, b). Modern high-intensity agriculture has a negative side effect of degrading agricultural land; some estimates suggest that more than one third of originally arable land has been degraded by human impacts (Oldeman, 1992). Returning organic carbon and nutrients from algal biomass to degraded agricultural land can potentially slow or reverse this trend, providing both food security and a sink for algal biomass. Biomass whose toxin concentration is unacceptable for agriculture or aquaculture must be processed to improve its quality if intended for food production. Such biomass can also be used for producing biofuels, other commodities or higher value products, or landfilled, with any toxic residues from further processing either landfilled or recycled.

If globally distributed throughout the world's river basins at large enough scale, increased algae cultivation could directly reverse the transgressions of planetary boundaries (Steffen et al., 2015) involving the N and P biogeochemical cycles. Here we estimate the scope of such an intervention both from a global perspective and by prioritizing major hydrological basins, as curated in publicly available nutrient and geospatial datasets. We discuss potential issues with land availability, economics, and sociopolitical considerations, ultimately extrapolating our analysis to complete recycling of net anthropogenic carbon. Our stance is conservative, as our intent is to determine an approximate upper limit to the effort needed to counteract these global threats and compare our proposed intervention with other global projects of similar scale. Improvements in biomass productivity and efficiencies in design, construction, and operation will reduce the investment required. Improving our model's accuracy and precision will require expanding current limited usage to a multi-year global program of building and operating pilot projects, affording development of a predictive productivity model and providing a more extensive knowledge base specific regional requirements.

## 2 Methods

Data processing was organized using R Studio 1.0.143 (RStudio Team, 2016), integrating R 3.4.2 (R Core Team, 2015) and various R packages; source code and instructions for reproducing our findings are available at http://github.com/calahan/EACCRPBT and http://github.com/calahan/Calahanlab. Model parameters are organized in the spreadsheet EACCRPBT.xlsx and imported as variables into the R code used to generate figures and tables. Datasets were obtained from Natural Earth (continents) (Natural Earth, 2015), the Global Runoff Data Center (GRDC) (basins, lakes, and rivers) (Global Runoff Data Centre, 2007a, b), and EarthStat (N and P nutrient loading) (West et al., 2014) (obtaining GRDC data requires completing an application form). An 8 GB Apple MacBook Pro/2.5 GHz Intel Core i5 running OS X 10.11.6 was used for all data processing.

We assume biomass mass ratios of 36%, 6.3%, and 0.87% for C, N and P respectively, derived from the stoichiometric ratios of algal biomass (Ebeling et al., 2006; Hillebrand and Sommer, 1999). We assume equatorial productivity of 9.1 × 10^1^ t ha^−1^ yr^−1^ (2.5 × 10^2^ T ac^−1^ yr^−1^), and at 40°, of 5.5 × 10^1^ t ha^−1^ yr^−1^ (1.5 × 10^2^ T ac^−1^ yr^−1^), deriving a linear relationship as our productivity model. CapEx and OpEx estimates are ultimately derived from actual construction and operation figures (Hoffman et al., 2017). Values for economic parameters were chosen to represent best and worst cases for CapEx ($2.5 × 10^5^ ha^−1^ ($1.0 × 10^5^ ac^−1^) and $1.0 × 10^6^ ha^−1^ ($4.0 × 10^5^ ac^−1^ respectively)), OpEx ($3.3 × 10^4^ ha^−1^ ($1.3 × 10^4^ ac^−1^) and $7 × 10^4^ ha^−1^ yr^−1^ ($2.8 × 10^4^ ac^−1^) respectively) and facility lifetime (20 yr for low CapEx and 40 yr for high CapEx, respectively). Best and worst case OpEx values were chosen to represent fully encumbered lower and higher end middle-class salaries in the US and additional contingent costs of 10% of CapEx (Table 2). The economic model creates an R data frame by populating the first row (year 1) with initial conditions then applies the model's spending growth formula (10% yr^−1^), to that row to produce the next row (year 2), repeating the procedure for year 3 to year 100. Constant spending is then applied for the following 100 yr. To determine the initial spending rate, we chose parameters such that once the system is complete, the minimum algal biomass is always sufficient to completely recycle the relevant nutrients.

**Table 2:** Parameters for Economic Model.

variable	value	unit
cap_lo	2.50 × 10^5^	$ ha^−1^
cap_hi	1.00 × 10^6^	$ ha^−1^
op_lo	3.30 × 10^4^	$ ha^−1^ yr^−1^
op_hi	7.00 × 10^4^	$ ha^−1^ yr^−1^
op_life_long	4.00 × 10^1^	yr
op_life_short	2.00 × 10^1^	yr
betterNP.exp	2.30 × 10^12^	$
betterNP.bm	3.10 × 10^9^	t yr^−1^
worseNP.exp	6.10 × 10^12^	$
worseNP.bm	3.10 × 10^9^	t yr^−1^
betterC.exp	1.60 × 10^13^	$
betterC.bm	2.20 × 10^10^	t yr^−1^
worseC.exp	4.30 × 10^13^	$
worseC.bm	2.20 × 10^10^	t yr^−1^

To identify the limiting nutrient for each grid cell with excess nutrients, we obtained mass ratios for the nutrients N and P by converting their stoichiometric Redfield ratios into mass ratios (R_N_, and R_P_ respectively), and obtained the Redfield P/N mass ratio, R_PN_ = R_P_/R_N_. Then for each grid cell we divided the excess P mass by the excess N mass to obtain its P/N mass ratio, GC_PN_, and compared this value to R_PN_, with locations having R_PN_ < GC_PN_ considered P limited and locations having R_PN_ ≥ GC_PN_ considered N limited. To determine the amount of algal biomass needed to incorporate the non-limiting nutrient for each grid cell, GC_BM_, we divided that grid cell's excess mass of that nutrient, M_Nut_, by R_N_ or R_P_, depending on the identity of the nutrient, B_gc_ = M_Nut_/R_Nut_. To determine the algal cultivation area needed to produce the required amount of biomass for each location, we modeled algal productivity as a linear function of latitude, with each grid cell considered as a trapezoid centered on its associated coordinates. To rank basins by investment required to recycle nutrient excesses, we then summed the algal growth area for each grid cell contained by each basin.

## 3 Results

**Fig. 2:**
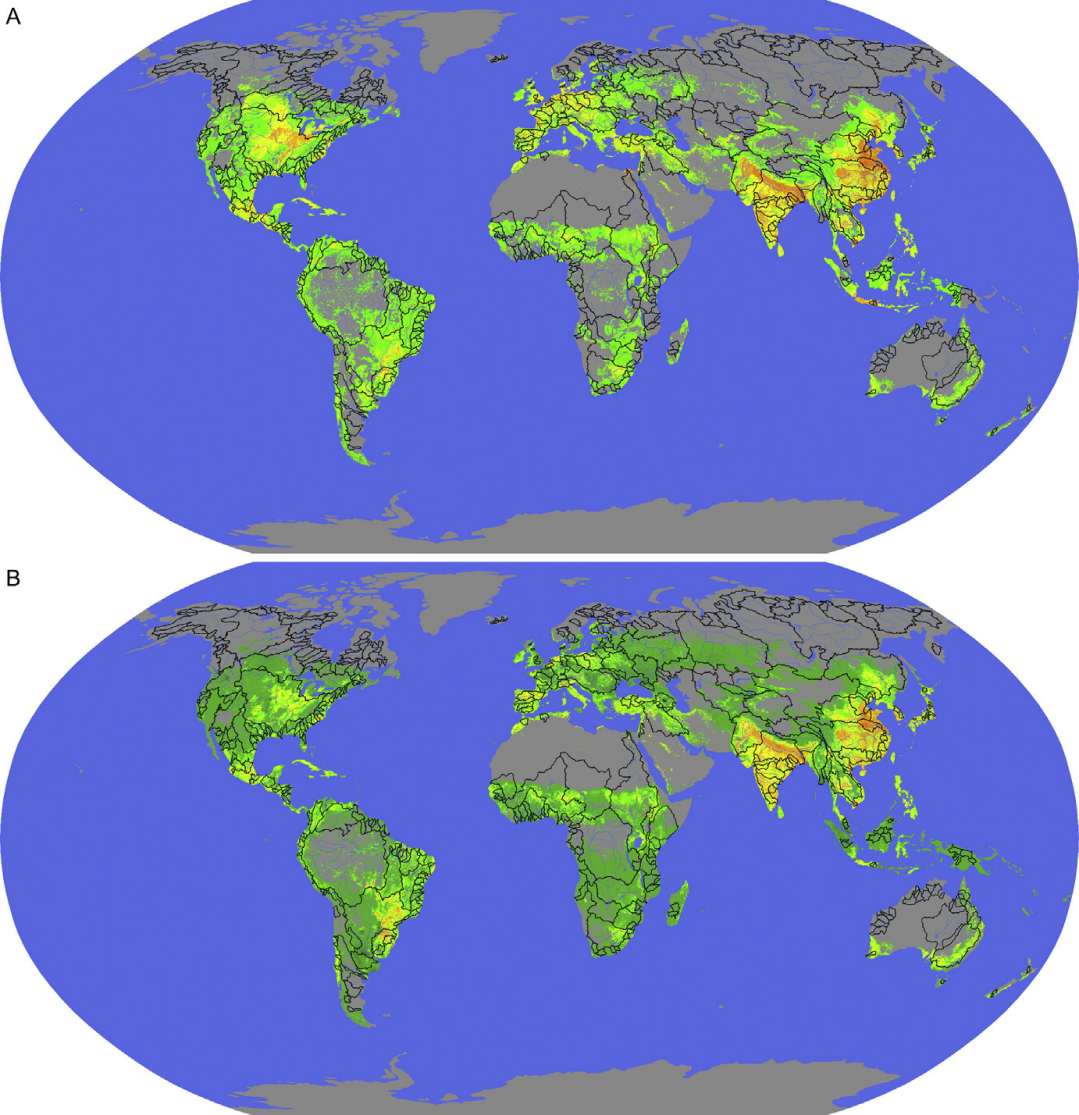
Global distribution of nutrient excesses and deficits. Gray indicates locations with no nutrient data. (A) N excess. Red shades indicate grid cells with the largest excesses, representing 10% of the total; orange-to-yellow shades indicate grid cells responsible for the next-highest 40% of total excess; green shades represent the lowest 50%. (B) P excess. Coloring scheme is as in A, but with the addition of darker green indicating grid cells with P deficit.

**Fig. 3:**
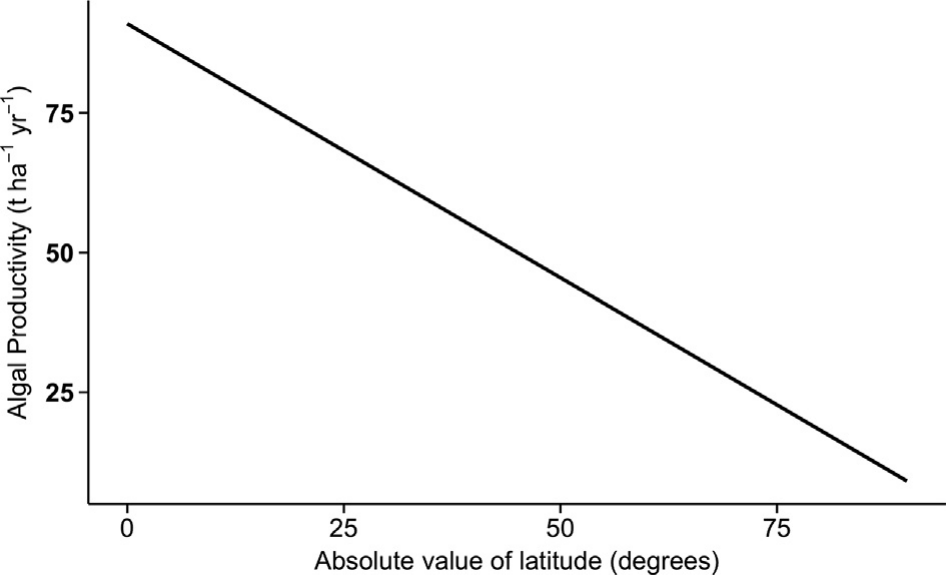
Relation of biomass productivity to degrees of latitude (north or south). Model was constructed by assigning 9.1 × 10^1^ t ha^−1^ yr^−1^ to 0° and 5.5 × 10^1^ t ha^−1^ yr^−1^ to 40° and deriving a linear relation.

Nutrient data (Potter et al., 2010) are organized on a 0.5′ × 0.5′ grid of latitude and longitude, with 9,318,244 total grid cells, 698,788 having both N and P data and 228,398 having P data only (Fig. 2). Values represent kg of excess nutrient per grid cell, and are positive for N but positive or negative for P. We consider only positive values, as nutrient deficits are not transferrable among grid cells or basins. Excess N per grid cell ranges from 0 kg to 2.8 × 10^6^ kg. Excess P per grid cell ranges from 0 kg to 5.8 × 10^5^ kg. Half of the total excess N and P is accounted for by 6.8% and 7.0% of their respective grid cells. The 405 major basins curated by the Global Runoff Data Center (Global Runoff Data Centre, 2007b) are responsible for 77% of global nutrient excess, for both N and P. Excess N per basin ranges from 0 to 5.1 × 10^9^ kg yr^−1.^ Excess P per basin ranges from 0 kg yr^−1^ to 1 × 10^9^ kg yr^−1^. Half of the nutrient excess is contributed by five (N) or six (P) basins. The Yangtze contributes the most N, while the Ganges contributes the most P (see Fig. 3, Table 3).

**Table 3:** Highest priority basins.

Basin Name	ATS Area (ha)	ATS Area (%, vs. basin)	ATS Area (%, vs. total)
Yangtze	1.80 × 10^6^	12.2	12
Ganges	1.70 × 10^6^	12.0	24
Mississippi	1.20 × 10^6^	8.1	32
Indus	8.50 × 10^5^	5.9	38
Huai	7.80 × 10^5^	5.4	44
Huang	7.20 × 10^5^	5.0	49
Parana	6.40 × 10^5^	4.5	53
Amur	3.40 × 10^5^	2.3	55
Yongding	3.30 × 10^5^	2.3	58
Xi Jiang	2.70 × 10^5^	1.9	60
Nelson	2.60 × 10^5^	1.8	61
Krishna	2.40 × 10^5^	1.7	63
Godavari	2.30 × 10^5^	1.6	65
Nile	2.00 × 10^5^	1.4	66
Mekong	2.00 × 10^5^	1.4	67

**Fig. 4:**
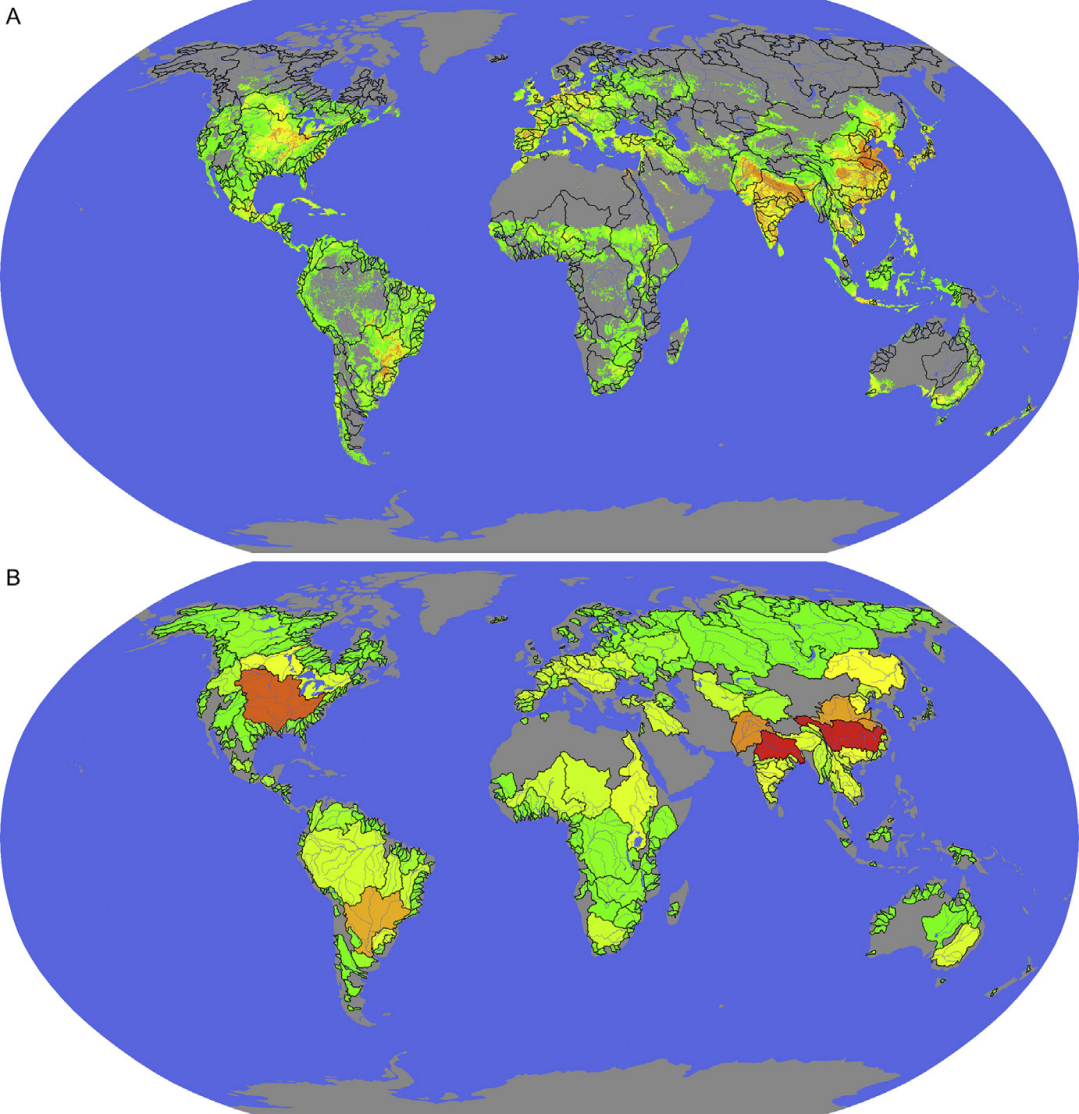
Algal cultivation required for nutrient recycling. (A) Grid cells ranked by algal cultivation area required to recycle each grid cell's non-limiting nutrient, red cells indicating the 10% requiring the highest cultivation area, yellow the next 40%, and green the lowest 50%. (B) Basins ranked by algal cultivation area required to recycle each basin's non-limiting nutrient, color scheme as in A.

Globally, excess N and P applied to the 140 crops curated in the EarthStat datasets (West et al., 2014) total 5 × 10^7^ t (5.5 × 10^7^ T) and 9.1 × 10^6^ t (1 × 10^7^ T), respectively (we abbreviate metric tons with ‘t’ and US tons with ‘T’). These excesses imply N-limitation in 405,453 grid cells and P-limitation in 293,335 grid cells. As complete recycling of a limiting nutrient in a given grid cell or basin would leave a surplus of the non-limiting nutrient, we compute the algal cultivation area required to recycle P in N-limited grid cells, and to recycle N in P-limited grid cells (Fig. 4A), summing these areas over each basin (Fig. 4B). Globally, 1.9 × 10^7^ ha (4.7 × 10^7^ ac) of algal cultivation area is required for complete N and P recycling. For nutrient recycling in the major basins curated in the GRDC data set, a total of 1.4 × 10^7^ ha (3.5 × 10^7^ ac) is required. The total biomass produced, 1.2 × 10^9^ t yr^−1^ (1.3 × 10^9^ T yr^−1^), is equivalent to 1.2% of world net primary productivity, or approximately 15% of world agriculture productivity. The fuel value of the carbohydrate and protein content of this quantity of ATS biomass (Pate, 2015) is approximately 5.0 × 10^11^ W, 2.8% of global primary energy estimated at 1.8 × 10^13^ W (IEA, 2017).

**Fig. 5:**
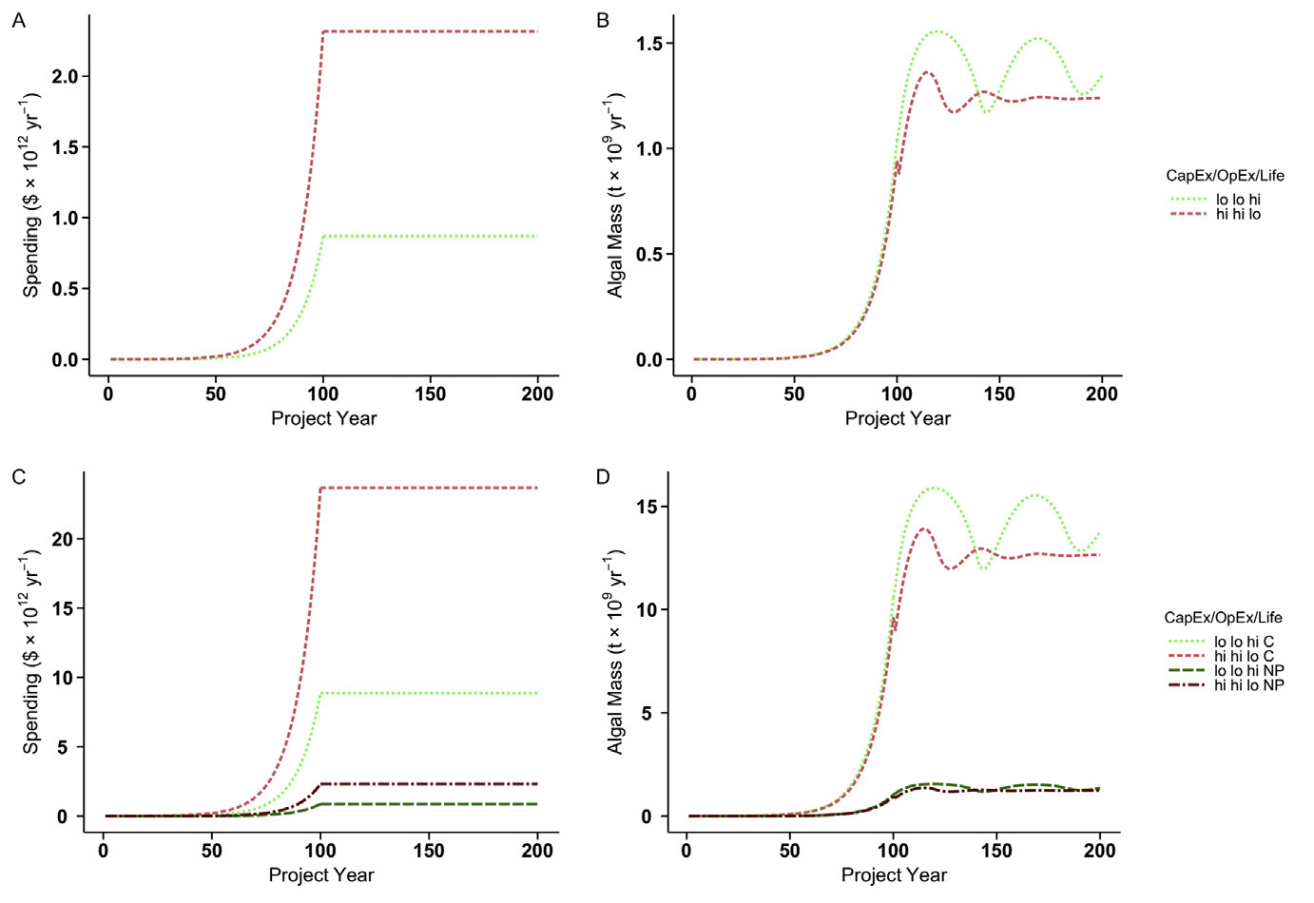
Gross expense and biomass production for recycling N/P and C. Two scenarios are illustrated: High CapEx/OpEx with a 20 year floway life expectancy (worst case; “hi hi lo”), and low CapEx/OpEx with a 40 year floway life expectancy (best case; “lo lo hi”). (A) Best and worst cases for N/P recycling eventually require spending $8.7 × 10^11^ and $2.3 × 10^12^ annually, respectively. (B) N/P recycling produces 1.2 × 10^9^ t yr^−1^ algal biomass. (C) Best and worst cases for C recycling require eventually spending $8.9 × 10^12^ yr^−1^ and $2.4 × 10^13^ yr^−1^, respectively. (D) C recycling produces, 2.2 × 10^10^ t yr^−1^ algal biomass. (C and D) N and P recycling included for scale.

Our economic model for N and P recycling assumes continued application of excess agricultural nutrients at the current rate. Our worst and best case scenarios bracket a range of potential expenses, determined by floway cost and lifetime (CapEx), and operator salary (OpEx). We adopt a simple investment scenario starting with an initial annual spending rate (best case: $6.9 × 10^7^ yr^−1^; worst case: $1.9 × 10^8^ yr^−1^), that increases by 10% each year for 100 years, then remains at that final level (best case: $8.7 × 10^11^ yr^−1;^ worst case: $2.3 × 10^12^ yr^−1^) for the next 100 years, setting the initial spending at a level that results in complete nutrient recycling after year 100 (Table 2, Fig. 5). This exponential spending increase, combined with the limited lifetime of an ATS facility produces the “ringing” phenomenon apparent in the plots of biomass production, while the sudden halt to investment growth at year 100 results in a discontinuity apparent as a change in slope of biomass production. We apply the same tactic to estimate the effort needed to recycle net anthropogenic C, finding the initial investment rate (best case: $7.1 × 10^8^ yr^−1^; worst case: $1.9 × 10^9^ yr^−1^) and final investment rate (best case: $8.9 × 10^12^ yr^−1^; worst case: $2.4 × 10^13^ yr^−1^) to be an order of magnitude greater than for N and P recycling.

## 4 Discussion

Here we find that a 100 year project to expand algal cultivation to completely recycle global agricultural N and P excesses would, when complete, require global gross expenses no greater than $2.3 × 10^12^ yr^−1^, 3.0% of the 2016 world gross domestic product of $7.6 × 10^13^, and commitment of less than 1.9 × 10^7^ ha (4.6 × 10^7^ ac) of land, 0.38% of the 4.9 × 10^9^ ha used globally to grow food. Thus the financial burden to achieve this environmental benefit appears tractable in terms of both expense and land use. Our upper limit assumes conservative economic parameters (high CapEx and OpEx), problem conditions (continued excess nutrient application at today's rate), and problem solution (in effect, a return to pre-industrial conditions of global freshwater drainage). In reality, the economics may not be as extreme as our worst case assumptions, may be more moderate when averaged globally, and may improve with increased experience and innovation. Improvements to agricultural practices may reduce the scale of the problem, and such a comprehensive solution may not ultimately be necessary. Thus it is likely that our worst case upper limit on the required effort, already apparently tractable, can be substantially reduced. Furthermore, we model gross expense without considering the economic and agronomic effects of the copious algal biomass produced as a consequence of nutrient recycling, which may offset expenses by providing a valuable commodity, reduce the scale of the problem by improving soils, and potentially even slow or reverse the loss of arable land. Because biomass production is also tightly coupled to the carbon cycle, we extrapolate our model to include recycling of net anthropogenic carbon, finding that the solution to this problem requires an order of magnitude greater investment. Again, our conservative assumptions imply the possibility of considerably smaller effort being required, especially if fossil fuel combustion is rapidly reduced.

Because we approximate the effort needed to globally recycle pollutants, rather than create a detailed simulation, we chose a round number (100 years) for our project duration, and a simple algorithm for spending: exponential growth starting at a defined spending rate and ending at a level that satisfies the desired pollutant recycling rate. This approach to financing and scheduling is rudimentary, but the large scale of our proposed intervention makes it practically impossible to incorporate all the various financing, permitting, and sociopolitical situations affecting jurisdictions of interest in advance of intensive economic engineering. Both the technology and the magnitude are similar to highway construction, supporting our tractability finding. Highway costs per unit area are similar to ATS floway costs (FHWA, 2015a, b), and the global extent of paved roads is much greater than the floway area we require. Another way to conceptualize the scale of our proposed intervention is to consider the potential fuel value of the carbohydrate and protein content of the produced biomass (Pate, 2015), 5.0 × 10^11^ W. This value is 2.8% of global primary energy estimated at 1.8 × 10^13^ W, representing an appreciable photosynthetic subsidy for ATS compared to other possible methods of cleaning nonpoint N and P pollution. Our linear model relating latitude to productivity ignores climatic differences along latitude lines, and can only be corrected by a comprehensive set of pilot studies, both to elucidate basin-by-basin differences in productivity, and to more completely define the design parameters that maximize it. Our ultimate spending rate is a substantial percentage of the world gross domestic product (The World Bank, 2017), suggesting that the commodity flow of algal biomass will affect markets for other commodities, potentially prompting resistance from affected parties, but we do not address these impacts. The basin component of our analysis focuses on 405 curated major watersheds. A more comprehensive analysis would consider the remaining basins, but visual inspection of Figs. 2 and 4 suggests that our prioritization would be little changed, especially regarding the basins with the greatest excesses. Fig. 2 also reveals patterns of lacunae (for example, an approximately Arizona-shaped void in western North America) suggesting that the nutrient data may be incomplete. However, even if formally incomplete, the coverage is nevertheless sufficiently extensive that our main conclusions would likely be little changed with more complete data.

Two complementary approaches for implementing our proposed intervention are available. As problems caused by excess nutrients become more frequent or severe, afflicted jurisdictions might increasingly undertake local implementation of ATS, as has already occurred in several locations (Table 1). The availability of bounties for nutrients might encourage private concerns to participate, or local taxes might be used to directly subsidize implementation, considering nutrient recycling to be a public good. If a market for algal biomass can be developed, revenue from that market can help offset expenses. In some basins, perhaps most, this natural economic growth in algae cultivation might be sufficient to contribute substantially to basin remediation. For basins with the greatest excess nutrient loads (Table 3), the required floway area can be a large proportion of the basin area, thus a more organized approach may be essential to ensure that each increment of investment achieves the highest nutrient removal rate. The current global ecological state derives from centuries of increasing human impact on ecosystem services; centuries of increasing countermeasures may be required to completely reverse the damage, with our proposed 100-year project but one component. It may thus be prudent to start with projects at scales affording early and definitive measures of success. Initially, only local effects will be easily discernible, so focusing much of the initial investment on a few high priority basins or sub-basins might deliver verifiable improvements rapidly and visibly. Beyond water and biomass quality measurements, initial measures of success could include reduction in the frequency, duration, and extent of HABs within affected waters, improved health of reef and other ecologies in the estuaries and bays receiving the discharge, and improved agricultural metrics where algal biomass is applied to the soil.

The scale of our proposed intervention prompts concerns about environmental impacts. For example, in highly industrialized basins algal biomass that concentrates toxins from polluted water may itself become toxic waste, requiring treatment to accommodate further uses. With landfilling the default fate of biomass for current production units, uses that both generate revenue and lower residue volume can improve the economics. Biofuel production could remove up to one third of the biomass as carbon, concentrating toxins in the residue and thus reducing landfill and transportation costs. Another possible impact is emissions from floways. Oxygen will be released during photosynthesis, both into the atmosphere and dissolved in the outflow, likely benefitting downstream ecosystems but also associated with increased pH potentially requiring partial neutralization. Many marine algae emit dimethyl sulfide (DMS), a volatile organosulfur compound that impacts climate by nucleating clouds over the oceans (Charlson et al., 1987). No studies of DMS emissions from ATS floways have yet been performed. Increased cloud cover could increase Earth's albedo, slowing global warming, but could also reduce agricultural and photovoltaic productivity. Other impacts that merit consideration relate to the algal cultivation area required. While algal floways are efficient at non-point nutrient removal on an areal basis, they nevertheless require conversion of land from current uses to algae cultivation. Non-agricultural land, brownfields, or even building roofs might be candidates for conversion, but where agriculture is most concentrated, existing crop production might have to be traded for algae production, requiring careful cost-benefit analysis. Wading birds are frequently observed in production floways, feeding on productivity-inhibiting algal grazers and adding additional nutrients to the water. This phenomenon will recur and increase with expanded algal cultivation, potentially affecting migration and transporting algal cells between ecosystems. These examples represent questions that will need to be investigated as algae cultivation expands from the few hectares now in production to the tens of millions of hectares needed.

## 5 Conclusions

We have shown that expanding algal cultivation using algal turf scrubbing (ATS) technology can recover excess global agricultural nutrients. The CapEx and OpEx required are large but tractable, and additional rewards would likely accrue through improving the global ecological state. The primary difficulties are probably not technological, but rather sociopolitical, with pluralities ever reluctant to commit resources to address affronts for which they are collectively responsible, and macroeconomic, with such a large injection of biomass into world trade likely to prompt resistance from entities having a vested interest in the status quo. While an independent market for the enormous annual biomass production required may not develop immediately, bounties for N and P capture, as well as for C capture, are one way to subsidize establishment and operation of a growing algal cultivation infrastructure. If biofuels or other commodities can be profitably manufactured from this algal biomass, subsidies can eventually be reduced or eliminated. One of the most cost-effective and beneficial uses for algal biomass may be returning it to local land as a soil amendment and fertilizer, potentially slowing or reversing the loss of arable land, delaying peak phosphorus, and reducing the energy and materials expended on manufacture of N fertilizer. This potential biomass sink is very deep, likely able to accept biomass for several centuries, as appreciable soil depletion has been occurring for millenia, with further agriculture and aquaculture applications also to be demonstrated. While additional efforts to either reduce nutrient application or reclaim excesses of nutrients may be implemented, to date none of these appear as promising as ATS for global deployment.

## Declarations

### Author contribution statement

Steven D. Calahan: Conceived and designed the experiments; Performed the experiments; Analyzed and interpreted the data; Contributed reagents, materials, analysis tools or data; Wrote the paper.

Edward Osenbaugh, Walter Adey: Conceived and designed the experiments; Analyzed and interpreted the data; Contributed reagents, materials, analysis tools or data.

### Funding statement

This research did not receive any specific grant from funding agencies in the public, commercial, or not-for-profit sectors.

### Competing interest statement

The authors declare the following conflict of interests: Walter Adey is president of Ecological Systems Technology, Inc., the owner of intellectual property licensed to HydroMentia Technologies, LLC, the major corporate interest involved with ATS.

### Additional information

## Appendix A Supplementary data

Supplemental content spreadsheet
